# Reliability and modality analysis of patellar height measurement in pediatric knee

**DOI:** 10.3389/fped.2024.1323015

**Published:** 2024-03-26

**Authors:** Yoon Hae Kwak, Soo-Sung Park, Aaron J. Huser, Keunho Kim, Yong-Gon Koh, Ji-Hoon Nam, Kyoung-Tak Kang

**Affiliations:** ^1^ Department of Orthopedic Surgery, Asan Medical Center Children's Hospital, University of Ulsan College of Medicine, Seoul, Republic of Korea; ^2^ Department of Orthopedic Surgery, Paley Advanced Limb Lengthening Institute, St. Mary’s Hospital, West Palm Beach, FL, United States; ^3^ Joint Reconstruction Center, Department of Orthopaedic Surgery, Yonsei Sarang Hospital, Seoul, Republic of Korea; ^4^ Department of Mechanical Engineering, Yonsei University, Seoul, Republic of Korea

**Keywords:** Korean pediatric patients, morphometry, patellar height, reliability, inter-modality

## Abstract

**Introduction:**

Various measurement methods and imaging technique are in use to measure patellar height in pediatric patients. However, there is no gold standard as to which measurement method and modality are the most reliable for pediatric patients. Therefore, the aim of this study was to determine the inter-observer reliability, intra-observer reliability, and applicability of various patellar height measurement methods in pediatric knee. Additionaly, we analyzed the reliability across different imaging modalities.

**Methods:**

Total 450 pediatric patients (age: 5–18 years) were evaluated using lateral knee radiographs and magnetic resonance imaging (MRI). The patellar height ratios were measured using five methods. Five methods were Insall–Salvati (IS), Koshino–Sugimoto (KS), Blackburne–Peel (BP), modified Insall–Salvati (MIS), and Caton–Deschamps (CD). The patients were categorized into two age groups: P (ages 5–13) and Q (ages 14–18). Each measurement was conducted twice by two raters. The intra-observer reliability, inter-observer reliability and inter-modality reliability were calculated. In addition, applicability was defined as the possibility to apply each measurement method to each age group.

**Results:**

The KS method showed the highest inter-observer reliability and intra-observer reliability when using MRI for both age groups. The inter-observer reliability and intra-observer reliability of the IS for lateral knee radiographs was highest among all observers for group Q. The CD method showed the highest inter-observer reliability in group P, while the KS showed the highest intra-observer reliability in group P using lateral radiographs. The KS method showed the highest inter-modality reliability in group P, while the IS showed the highest inter-modality reliability in group Q. The KS method was applicable to all patients when using lateral knee radiography, and the IS method was applicable to all patients when using MRI.

**Conclusions:**

Our results show that the reliability of various measurement method and imaging technique differed based on pediatric knee age group when measuring patellar height. Therefore, in the case of pediatric patients, reliability measurement methods and imaging techniques according to the patient's age should be applied.

## Introduction

Measurement of patellar height is important in evaluating patello-femoral (PF) instability and PF joint pain (1, 2). The measured patellar height becomes an important indicator when establishing a treatment plan for PF joint pain and instability (3). In general, patellar height is evaluated using lateral radiographs and sagittal plane imaging, which classifies the measurement as normal, patella alta, or patella baja (4). Multiple indices have been defined to measure the height of the patella.

The methods that utilize indirect assessment, such as the Insall–Salvati (IS), Koshino–Sugimoto (KS), Blackburne–Peel (BP), modified Insall–Salvati (MIS), and Caton–Deschamps (CD) have been used (5–8). However, most of these methods rely on bone landmarks, which may not be applicable to pediatric patients whose ossification is not complete (9, 10). The widely used imaging modalities for measuring patellar height include lateral radiographs, computed tomography (CT), and magnetic resonance imaging (MRI). The reliability of patellar tendon measurement on MRI is improved compared to radiographs because it can more clearly display patellar tendon, and PF cartilage (10, 11).

In pediatric patients, data on modalities other than lateral radiographs is limited, and it is often reported in combination with adult data (12, 13). Moreover, there is a focus on the IS method when measuring patellar height in pediatric patients (12, 13). Park et al. compared MRI and lateral radiographs to evaluate the most suitable method among the IS, BP, and KS techniques for measuring patellar height in pediatric patients (10). However, conducting MRI to measure patellar height in all pediatric patients is challenging (9). MRI is less cost-effective compared to radiographs and may require sedation for pediatric patients (9, 13). Recently, Kwak et al. used lateral radiographs to demonstrate differences in the reliability, variability, and applicability of patellar height measurement methods according to age groups in pediatric patients (9). According to their findings, age group and sex influence the choice of methods for patellar height measurement in pediatric patients (9). However, currently, there is no gold standard measurement method for measuring patellar height in pediatric patients.

Therefore, the aim of this study was to determine the inter-observer reliability and intra-observer reliability of various patellar height measurement methods in pediatric patients. In addition, the imaging inter-modality reliability was compared in pediatric patients to assess universal acceptance. This study also assessed which measurement methods were most applicable for different age groups. We hypothesized that different measurement methods and modalities should be used for patellar height measurement in pediatric patients based on age groups.

## Material & methods

### Patients

This study was approved by the hospital's institutional review board. The pediatric patients were retrospectively selected from the record of radiology department. The pediatric patients aged 5–18 years and both lateral knee radiographs and MRI were included. To evaluate patellar height in pediatric population, at first we recruited all the knee MRIs matched with lateral knee radiographs. We identified 580 pediatric patients between January 2012 and September 2022. Then we excluded patients with obvious knee deformities. The exclusion criteria were overall knee disorders as congenital deformities and tumorous condition and trauma as ligament injuries, fractures, previous knee surgeries based on the retrospective review of radiologic studies (10). We also excluded radiographs that deviated excessively from the true lateral view by more than 30° of flexion or rotation (9). After applying the exclusion criteria, a total of 450 pediatric patients (450 knees; 227 males and 223 females) were included in this study. The patients were divided into two groups: Group P (age: 5–13 years) and Group Q (age: 14–18 years) (Table 1) (14, 15).

**Table 1:** Comparison of the age and gender ratio between Korean pediatric normal and injury.

Parameter	Whole patients (*n* = 450)	Group P (*n* = 300)	Group Q (*n* = 150)
	Mean ± SD (range)	Mean ± SD (range)	Mean ± SD (range)
Age	11.9 ± 3.5 (5, 18)	10.0 ± 2.5 (5, 13.8)	15.9 ± 1.5 (14, 18)
Female/male	227/223	151/149	76/74

n.s, non-significant.

### Image acquisition

Patella height ratios were measured using lateral knee radiographs with a knee flexion of 30°. Radiographs were obtained using a GE Definium 8000 instrument. MRI scans were obtained using a 3.0-Tesla MRI scanner (Philips Medical Systems, Amsterdam, Netherlands). Imaging was performed as previously described (9).

### Measurements

**Figure 1:**
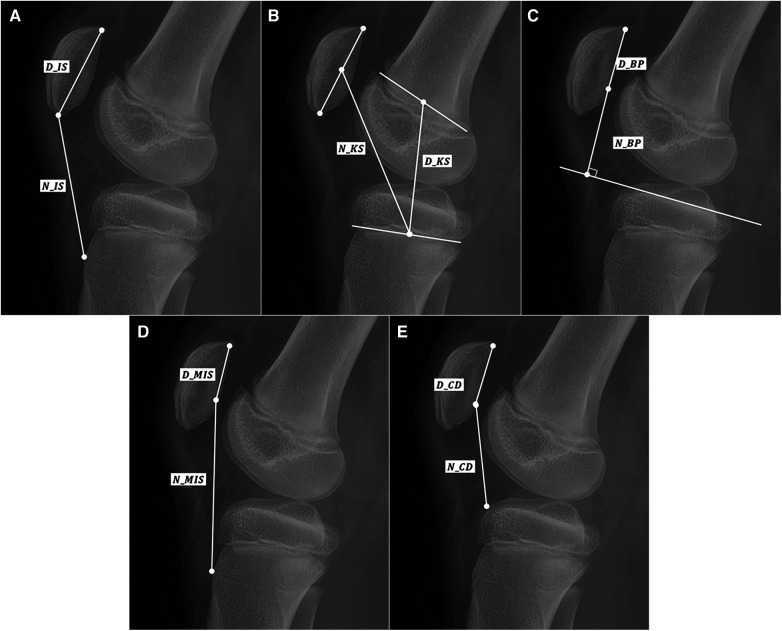
Schematic representation of (**A**) IS ratio, (**B**) KS ratio, (**C**) BP ratio, (**D**) MIS ratio, and (**E**) CD ratio mearued in MRI. (*D* and *N* indicate the denominator and numerator, respectively).

**Figure 2:**
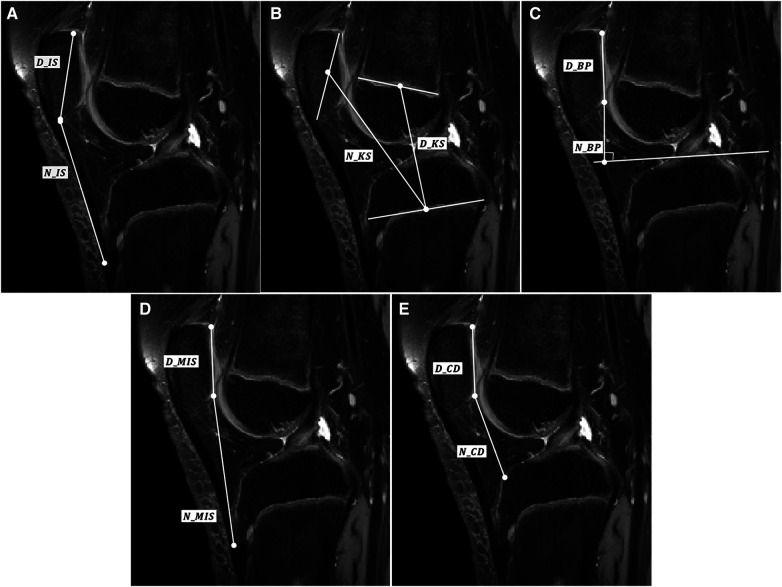
Schematic representation of (**A**) IS ratio, (**B**) KS ratio, (**C**) BP ratio, (**D**) MIS ratio, and (**E**) CD ratio mearued in lateral knee radiographs.

The patellar height ratios were measured using five methods. Five methods were IS, KS, BP, MIS, CD. The measurement was performed using the Mimics version 17.0 software (Materialise, Leuven, Belgium). Figures 1A, 2A show the IS ratio (N_IS/D_IS) for measuring patellar height on x-RAY and MRI. The IS ratio was calculated as the ratio of N_IS(patella tendon length) to D_IS(patella length). Figures 1B, 2B show the KS ratio (N_KS/D_KS) [7]. The KS ratio was calculated as the ratio of N_KS (the distance between the midpoint of the patella and the midpoint of the proximal tibial physis) to D_KS (the distance between the midpoint of the femoral distal physis and the midpoint of the proximal tibial physis) [17]. Figures 1C, 2C show the BP ratio (N_BP/D_BP). The BP ratio was calculated as the ratio of N_BP (the distance between the inferior edge of patellar articular surface and the tibial plateau line) to D_BP (the length of the patellar articular surface) (5). Figures 1D, 2D show the MIS ratio (N_MIS/D_MIS). The MIS ratio was calculated as the ratio of N_MIS (distance between the inferior edge of the patellar articular surface and the patellar tendon attachment on the tibia) to D_MIS(the length of the patellar articular surface) (6). Figures 1E, 2E show the MIS ratio (N_CD/D_CD). The CD ratio was calculated as the ratio of N_CD (the distance between the inferior edgeof the patellar articular surface and the anterior most point of the tibial plateau) to D_CD (the length of the patellar articular surface) (16). The measurement on MRI was performed on the slice showing the greatest length of the patella. Only in the BP method, tibial plateau line was measured on the slice showing medial plateau.

Each measurement was conducted twice by two raters. The intra-rater reliability, inter-rater reliability and inter-modality reliability were calculated without sampling. Because the patients were pediatric with incomplete ossification, some methods were not applicable. Applicability was defined as the possibility to apply measurement at each age. Applicability of each method was calculated and compared using regression curve and contingency table. The modality-based difference of applicability was also analyzed.

### Statistical analysis

Descriptive statistics were calculated. Inter-rater, intra-rater and inter-modality reliability were determined using intraclass correlation coefficients (ICC). Applicability was determined using logistic regression. All statistics were calculated using R 4.2.2.

## Results

When using MRI, the inter-observer reliability was highest for the KS method (0.86) and lowest for the BP method (0.33) in group P (Table 2). In group Q, the inter-observer reliability was highest for the KS method (0.79) and lowest for the BP method (0.31). The inter-observer reliability for the IS method and MIS method increased with age, while the remaining methods showed a decrease in inter-observer reliability. The KS method showed the highest inter-observer reliability in group P (0.95) and Q (0.91). The MIS method showed the lowest inter-observer reliability in group P (0.51) and Q (0.63). The intra-observer reliability for the CD method and MIS method increased with age, while the remaining methods showed a decrease in intra-observer reliability. When using lateral knee radiography, the inter-observer reliability was highest for the CD method (0.86) in group P, while in group Q, the IS method (0.90) showed the highest inter-observer reliability (Table 3). In terms of intra-observer reliability, the KS method (0.90) showed the highest in Group P, while the IS method (0.84) showed the highest in Group Q (Table 4, 5).

**Table 2:** Inter-observer reliability of patellar height ratios on MRI.

Group	Observers	IS		BP		CD		MIS		KS	
		ICC	95% CI	ICC	95% CI	ICC	95% CI	ICC	95% CI	ICC	95% CI
P	First	0.61	0.51–0.69	0.31	0.09–0.48	0.34	0.21–0.45	0.39	0.35–0.53	0.90	0.81–0.94
	Second	0.53	0.17–0.72	0.35	0.22–0.46	0.47	0.18–0.65	0.36	0.26–0.46	0.84	0.57–0.92
	Overall	0.56	0.36–0.69	0.33	0.17–0.45	0.40	0.24–0.53	0.39	0.31–0.45	0.86	0.70–0.92
Q	First	0.65	0.55–0.73	0.32	0.05–0.52	0.38	0.08–0.59	0.49	0.34–0.62	0.77	0.46–0.89
	Second	0.58	0.22–0.76	0.29	0.09–0.47	0.35	0.00–0.58	0.35	0.19–0.49	0.73	0.27–0.87
	Overall	0.61	0.51–0.69	0.31	0.09–0.48	0.36	0.05–0.58	0.43	0.31–0.53	0.79	0.39–0.88

**Table 3:** Inter-observer reliability of patellar height ratios on x-RAY.

Group	Observers	IS		BP		CD		MIS		KS	
		ICC	95% CI	ICC	95% CI	ICC	95% CI	ICC	95% CI	ICC	95% CI
P	First	0.77	0.72–0.81	0.69	0.63–0.75	0.86	0.79–0.91	0.73	0.60–0.81	0.84	0.05–0.95
	Second	0.81	0.67–0.88	0.85	0.79–0.88	0.86	0.82–0.89	0.73	0.67–0.78	0.83	0.19–0.94
	Overall	0.78	0.73–0.82	0.77	0.73–0.80	0.86	0.82–0.89	0.72	0.67–0.77	0.83	0.12–0.94
Q	First	0.90	0.87–0.93	0.71	0.50–0.82	0.84	0.78–0.88	0.63	0.37–0.78	0.76	0.04–0.92
	Second	0.89	0.62–0.95	0.79	0.62–0.88	0.82	0.72–0.89	0.84	0.78–0.88	0.78	0.29–0.91
	Overall	0.90	0.83–0.93	0.75	0.57–0.84	0.83	0.79–0.87	0.73	0.62–0.80	0.77	0.16–0.91

**Table 4:** Intra-observer reliability of patellar height ratios on MRI.

Group	Observers	IS		BP		CD		MIS		KS	
		ICC	95% CI	ICC	95% CI	ICC	95% CI	ICC	95% CI	ICC	95% CI
P	First	0.67	0.55–0.75	0.52	0.42–0.61	0.66	0.60–0.72	0.34	0.23–0.43	0.95	0.94–0.96
	Second	0.89	0.86–0.92	0.77	0.71–0.81	0.69	0.63–0.75	0.72	0.66–0.77	0.94	0.91–0.96
	Overall	0.76	0.72–0.79	0.68	0.63–0.72	0.71	0.67–0.75	0.51	0.45–0.57	0.95	0.94–0.96
Q	First	0.63	0.28–0.79	0.52	0.39–0.63	0.66	0.56–0.74	0.58	0.55–0.74	0.88	0.84–0.91
	Second	0.89	0.85–0.92	0.75	0.67–0.81	0.76	0.69–0.82	0.65	0.55–0.74	0.94	0.91–0.95
	Overall	0.71	0.60–0.79	0.66	0.59–0.72	0.75	0.69–0.79	0.63	0.55–0.69	0.91	0.89–0.93

**Table 5:** Intra-observer reliability of patellar height ratios on x-ray.

Group	Observers	IS		BP		CD		modIS		KS	
		ICC	95% CI	ICC	95% CI	ICC	95% CI	ICC	95% CI	ICC	95% CI
P	First	0.82	0.77–0.85	0.74	0.68–0.79	0.88	0.85–0.90	0.85	0.81–0.88	0.93	0.89–0.96
	Second	0.70	0.62–0.76	0.86	0.82–0.88	0.87	0.83–0.90	0.79	0.74–0.83	0.87	0.53–0.95
	Overall	0.74	0.70–0.77	0.79	0.76–0.82	0.87	0.85–0.89	0.82	0.79–0.85	0.90	0.77–0.95
Q	First	0.95	0.83–0.98	0.75	0.65–0.83	0.84	0.79–0.88	0.77	0.60–0.85	0.91	0.88–0.93
	Second	0.86	0.72–0.92	0.81	0.74–0.86	0.80	0.73–0.85	0.82	0.76–0.87	0.78	0.23–0.91
	Overall	0.90	0.88–0.92	0.78	0.71–0.83	0.82	0.78–0.85	0.79	0.71–0.85	0.84	0.70–0.91

Table 6 presents the ICCs for evaluating the inter-modality reliability of each measurement method. In P group, the KS method showed demonstrated the highest inter-modality reliability, while the IS method showed the lowest inter-modality reliability. However, in group Q, the IS method showed the highest inter-modality reliability. The applicability of the lateral radiograph in group P for the BP, IS, MIS, CD, KS methods were 55.2%, 49.2%, 38.1%, 55.2%, and 99.7%, respectively. In The applicability of the lateral radiograph in group Q for the BP, IS, MIS, CD, KS methods were 100%, 100%, 100%, 100%, and 100%, respectively. The applicability of the MRI in group P for the BP, IS, MIS, CD, KS methods were 56.5%, 99.7%, 56.5%, 56.5%, and 98.7%, respectively. The applicability of the MRI in group Q for the BP, IS, MIS, CD, KS methods were 100%, 100%, 100%, 100%, and 49.7%, respectively.

**Table 6:** Inter-modality reliability between MRI and x-ray.

Group	Observers	IS		BP		CD		modIS		KS	
		ICC	95% CI	ICC	95% CI	ICC	95% CI	ICC	95% CI	ICC	95% CI
P	First	0.02	−0.06 to 0.10	0.07	0.00–0.15	0.22	0.14–0.30	0.11	0.03–0.18	0.30	0.22–0.37
	Second	0.13	0.05–0.21	0.28	0.20–0.36	0.23	0.08–0.37	0.28	0.19–0.36	0.32	0.24–0.39
	Overall	0.09	0.03–0.14	0.13	0.07–0.18	0.25	0.16–0.33	0.19	0.13–0.25	0.33	0.28–0.38
Q	First	0.52	0.41–0.60	0.09	−0.01 to 0.19	0.26	0.15–0.36	0.27	0.14–0.40	0.33	0.22–0.43
	Second	0.62	0.53–0.70	0.25	0.14–0.35	0.21	0.00–0.39	0.24	0.07–0.38	0.41	0.31–0.50
	Overall	0.56	0.48–0.63	0.10	0.02–0.18	0.25	0.12–0.36	0.27	0.13–0.39	0.40	0.32–0.47

## Discussion

The most significant finding of this study was that the inter-observer reliability and intra-observer reliability of patellar height measurement methods varied depending on the age group.

Moreover, the reliability of patellar height measurement methods can differ across age groups depending on the imaging modality used. Therefore, the applicability of patellar height measurement methods vary depending on the age group.

Previously, researchers tried to validate the reliability of methods for measuring patellar height of pediatric population (17, 18). However, in the case of pediatric patients, these methods may not always be applicable due to the incomplete ossification of bony landmarks.

In the past decade, three notable studies have been conducted to determine reliable patella height measurements in pediatric patients (9, 10, 12). Park et al. conducted a study to determine the most suitable method for measuring patellar height in pediatric patients (10). They considered the applicability, concurrent validity, and reliability of different measurement techniques specific to these age groups (10). In terms of applicability, validity, and reliability, the study found that the IS method was superior in patients older than 13 years (10). However, their study has three limitations. Firstly, they conducted the research with a small sample size of only 108 patients. Secondly, they assumed the IS method based on MRI as the gold standard for validity analysis rather than plain radiograph. Lastly, they did not consider the CD method and MIS method in their study.

Recently, Kurowecki et al. conducted a study investigating whether the IS ratio and patella alta, as determined using MRI, are comparable to those determined using lateral knee radiography in pediatric patients (12). Their results showed a strong correlation between the IS ratio and patella alta measured using both MRI and radiographs in children aged 7.5 years and older (12). However, their study has a significant limitation as it was conducted with a very small sample size of only 49 patients. This limited sample size undermines the generalizability and reliability of the conclusions drawn from the study. Kwak et al. recently conducted a study to evaluate the interobserver and intra-observer reliabilities of five patellar height measurement methods (IS, KS, MIS, BP, and CD) in the assessment of lateral knee radiographs in 425 pediatric patients [9]. The study also aimed to determine which measurement methods were most suitable for specific sex and age groups (9). They concluded that different methods for measuring patellar height should be utilized based on the age group and sex of pediatric patients (9). However, their study was limited to lateral knee radiographs. While lateral knee radiographs have cost-effective advantages compared to MRI, it is important to note that MRI offers additional benefits such as the ability to display patellar tendon length and patellofemoral cartilage. Therefore, in measuring patellar height in pediatric patients with incomplete ossification, MRI may be more suitable.

The goal of this study was to determine the most suitable method for measuring patellar height in pediatric patients. We used inter-observer reliability, intra-observer reliability, imaging modality reliability, and applicability. The study investigated the five most commonly used methods for determining patellar height: IS, KS, MIS, BP and CD. Furthermore, the study divided the pediatric patients into only two age groups, which differs from previous studies that examined patellar height measurements (9, 10). By reducing the number of age groups from three to two, orthopedic surgeons can achieve a more efficient and timely diagnosis.

The IS ratio is a frequently used index for evaluating patellar height in adult patients (12). The IS ratio was initially validated using lateral knee radiographs taken at 30° of flexion in adults. The subsequent studies have investigated its application on computed tomography (CT), MRI, and ultrasound in adult patients (19, 20). The IS ratio has shown good reliability in measuring patellar height in pediatric patients compared to other methods (9, 10, 12). In the previous two studies demonstrated that the IS ratio had the highest reliability in pediatric patients aged 13 years and older (9, 10). Another study suggested that it can be used to diagnose patella alta in patients aged 7.5 years and older (12). These findings align with the results of our study.

Our study found that when using lateral knee radiography, the IS method demonstrated the highest inter-observer and intra-observer reliability in pediatric patients aged 14 years and older. In a previous study, which examined the inter-observer reliability of patellar height measurements using lateral knee radiography, it was found that CD had better reliability compared to BP and IS (21). However, in the current study with patients aged 14 and older, the IS method demonstrated the highest intra-observer reliability compared to CD, BP, KS, and MIS. This finding is in contrast to the previous study. This was not only the case for lateral knee radiography but also for CT and MRI. The previous study also suggested that the intra-observer reliability of a measurement method may be related to rater's experience (21), and another study showed similar results (22).

Interestingly, the reliability of the IS method in the Q group when using MRI did not show the same reliability. In the MRI study, both the P and Q groups demonstrated that the KS method exhibited the highest inter-observer and intra-observer reliability. The KS measurement is the only method that does not use the length of the patella to determine its ratio. However, as the slices change, the shape of the patella can also vary, leading to changes in its length. Therefore, methods that utilize the patellar dimensions are more susceptible to the influence of slice selection in MRI. On the other hand, the KS method uses the position of the centroid instead of the length. The centroid position is less affected by changes in slices compared to the length measurement. This is why we believe the KS method demonstrates higher reliability in MRI compared to other methods.

Verhulst et al. also reported that different observers might select different sagittal slices on different occasions, which can result in decreased intra- and inter-observer reliability (21). Our results for imaging inter-modality reliability demonstrated that IS method in the Q group was the highest. Previous studies have also shown that in patients aged 14 and older, the IS method exhibited the highest inter-modality reliability (10, 22). In our study, when using lateral knee radiography, only the KS method demonstrated an applicability of over 99%. This is consistent with previous research findings (9, 10) indicating that the KS method might be a reliable and widely applicable method for measuring patellar height in lateral knee radiography.

The KS method is the only technique that utilizes the distance between the distal femoral physis and proximal tibial physis. Unlike other methods, it does not rely on the further ossification of the tibial tuberosity or tibial plateau (9, 10). However, when using MRI, the applicability of the KS method decreases by approximately 50%. The reason distal femoral physis is not well visualized when using MRI is because of the imaging technique itself. MRI utilizes a strong magnetic field and radio waves to generate images, which have a better interaction with soft tissues and fluids. However, bone tissue generates a weaker signal in response to the magnetic field, causing bone structures to appear relatively darker on MRI. In contrast, the IS method, which showed an applicability of approximately 50% when using lateral knee radiography in the P group, increased to 100% when using MRI. The patellar tendon, which was not visible in lateral knee radiography, is clearly visible in MRI. This explains why the applicability of the IS method increased to 100% when using MRI.

Our results demonstrate variations in inter-observer reliability and intra-observer reliability based on age groups. Additionally, there are differences in inter-modality reliability among different age groups. Our findings indicate that the applicability varies among age groups depending on the measurement method and imaging modality. This study has several limitations. First, while our study had a larger sample size of pediatric patients compared to previous studies (9, 10, 12), it is important to acknowledge the limitation in terms of ethnic diversity. This suggests a need for future research to include a more diverse representation of participants in future research. Second, due to the retrospective nature of this study, the degree of knee flexion on lateral knee radiographs may not be consistent with that observed on MRI [9–10]. Third, we used same age threshold between male and female patients in spite of different patella ossification age. Further larger-scale analysis should be conducted based on this consideration.

## Conclusions

Our results show that the reliability according to the measurement method and imaging technique differs according to the pediatric knee age group when measuring patellar height. Therefore, in the case of pediatric patients, the application of measurement methods and imaging techniques should be tailored to the patient's age. This has important clinical implications in the assessment of pediatric patellofemoral instability and it unscores the importance of methodological considerations in clinical evaluations.

## Data Availability

The raw data supporting the conclusions of this article will be made available by the authors, without undue reservation.
